# Effects of Species, Post-Harvest Treatment, and Roasting on Fibre, Volatile Compounds, and Polyphenol Contents in Coffee Silverskin

**DOI:** 10.3390/foods11193132

**Published:** 2022-10-08

**Authors:** Manuela Giordano, Marta Bertolino, Simona Belviso, Daniela Ghirardello, Giuseppe Zeppa

**Affiliations:** Department of Agricultural, Forest and Food Sciences (DISAFA), University of Torino, 10095 Grugliasco, Italy

**Keywords:** coffee silverskin, washed coffee, natural coffee, volatile compounds, polyphenols, antioxidants, roasting

## Abstract

Although coffee silverskin (CS) has recently been used as a food ingredient, no knowledge has been reported on the effects of species or different post-harvest treatments on its chemical composition. Therefore, the fibre, volatile compounds, phenolic acid content, and antioxidant capacity of CS samples obtained at three roasting intensities (light, medium, and dark) from the *Coffea arabica* and *C. canephora* species, each subjected to a washing or a sun-drying (“natural”) post-harvest treatment, were studied. Obtained results showed that the chemical composition of CS is due to species, roasting, post-harvest treatment, and interaction. In particular, natural *Arabica* CS showed the highest content of volatile compounds of Maillard and varietal origin, whereas washed *Arabica* CS showed the highest content of soluble dietary fibre and chlorogenic derivatives. Pyrroles, sulphur compounds, and pyridines contents were higher in *Canephora* CS than in *Arabica* CS. The dark-roasted washed *Arabica* CS showed the highest content of 5-*O*- and 3-*O*-caffeoylquinic acids, while the natural *Arabica* CS highlighted the highest antioxidant capacity. The effect of post-harvest treatments seemed to be emphasised in *Arabica* CS, independent of roasting, which did not significantly affect the antioxidant capacity of CS from either species.

## 1. Introduction

Coffee is an important commercial crop with a worldwide production of approximately 10.5 million tons in 2020–2021. In the future, *Arabica* (*C. arabica* L.) production is expected to increase by 13.7% to 105 million 60 kg bags, whereas a 3.2% reduction is expected in the production of Robusta (*C. canephora* Pierre ex Froehner) coffee to 70.1 million bags [1]. During coffee production, coffee cherries are subjected to two different post-harvest treatments: natural (sun-drying) and wash (wet or pulped). Natural/dry processing is more sustainable than wet processing because of its low water consumption and lower production of organic residues. The whole, intact, mature coffee cherries (beans, mucilage, and pulp) are dried in the sun or using dryers, normally at temperatures of up to 45 °C. However, the dry method is a time-consuming process, and the elevated moisture and sugar content of the mucilage increases the risk of undesirable fermentation [2,3]. A lack of care during harvest can then lead to the production of low-quality natural coffee. In the wet process, which generally results in higher quality coffee, ripe cherries are subjected to peeling and depulping mechanical processes. Residual mucilage is removed by controlled fermentation in the presence (wet fermentation) or absence (dry fermentation) of water. The coffee is washed to completely remove residual mucilage and then dried [2,4]. Subsequently, in both processes, the green coffee is subjected to roasting that is performed at different temperatures to generate aromatic compounds, which characterise the roasted coffee beans. During the roasting process, when the beans expand, the thin tegument of the outer layer of coffee beans, also known as coffee silverskin (CS), representing approximately 4% (*w*/*w*) of the coffee beans, is detached with a world production of about 0.4 million tons. CS is the main by-product of the coffee industry and in recent years, different characterisation studies on the bulk CS of undefined species have been conducted. The composition of this by-product shows a high dietary fibre content (56%), protein (18.8%), and minerals (almost 8.5% of ash). Potassium, magnesium, and calcium are the most abundant macrominerals, followed by Fe and Na. The total fat content is approximately 2.4%, whereas carbohydrates account for 5.8% of the chemical composition [5,6,7]. In addition, mandatory safety assays conducted on CS show the absence of pesticides and mycotoxins, and mitigation of other contaminants such as acrylamide [8]. CS is also characterised by the presence of caffeine, tannins, and melanoidins formed through the Maillard reaction during roasting [9,10,11,12], as well as phenolic compounds and alkaloids with a high antioxidant capacity [13].

Although CS has been mostly used as a combustible or fertiliser [14], the authors have suggested the use of CS as an important food ingredient that can be incorporated in several food formulations [15,16,17]. CS can be used as a raw food ingredient [18,19,20,21,22], but also as an extract to improve the quality of food [23,24,25,26] and beverages [27].

Despite these applications, to our knowledge, no further studies have been performed on the synergistic effects of coffee species (*Arabica* or *Canephora*), post-harvest treatment (wash/wet or natural/dry), and roasting intensity on the chemical composition of CS. These aspects have been largely examined for raw and roasted coffee beans, showing significant effects on all chemical parameters [28,29], aroma compounds [30,31,32,33], and polyphenol contents [34,35].

The present study aimed to explore the effects of natural and washing processes and three roasting conditions on fibre, volatile compounds, and phenolic acid contents, as well as antioxidant activity of CS from *C. arabica* and *C. canephora*.

## 2. Materials and Methods

### 2.1. Reagent and Standards

*n*-Hexane, acetone, ethanol, methanol, formic acid, trans-5-*O*-caffeoylquinic acid (*trans*-5-CQA), 2,2-diphenyl-1-picrylhydrazyl (DPPH), (±)-6-hydroxy-2,5,7,8-tetramethylchromane-2-carboxylic acid (Trolox), 2,2′-azobis(2-methylpropionamidine) dihydrochloride (AAPH), 3′,6′-dihydroxyspiro[isobenzofuran-1[3H],9′[9H]-xanthen]-3-one (fluorescein), Folin–Ciocalteu’s phenol reagent, and α-thujone were purchased from Sigma-Aldrich (Milan, Italy). All the chemicals used were of gas chromatography (GC)- and high-performance liquid chromatography (HPLC)-reagent grade. Ultrapure water was produced using a Milli-Q system (Millipore, Milan, Italy).

### 2.2. Raw Materials

CS was provided by a local coffee producer (Casa del Caffè Vergnano Spa, Santena (TO), Italy). Green coffee beans of *C. arabica* L. and *C. canephora* Pierre ex Froehner were obtained from two post-harvest treatments, washed/wet and natural/dry, and subjected to three industrial roasting treatments with a drum coffee roaster (Petroncini Impianti S.p.A., Sant’Agostino, Italy) in a batch weighing 30 kg. The roasting degree of the coffee was expressed in Pt Colorette values, using a Probat Colorette 3b (Probat-Werke von Gimborn Maschinenfabrik GmbH, Emmerich am Rhein, Germany), according to a coffee colorimetric standardised scale of 0–200, which is related to luminance L* values in the CIE colour system. The coffee was roasted at an average colorimetric value of 110 ± 5 for light, 93 ± 4 for medium, and 84 ± 1 for dark.

The CS was ground using a laboratory grinder Retsch ZM200 (Retsch GmbH, Haan, Germany), separated according to a particle size of 250 μm using a 60-mesh sieve, and stored at 20 °C in sealed polyethylene plastic bags until analysis.

### 2.3. Dietary Fibre

Total, insoluble, and soluble fibre contents were determined using the AOAC 991.43 method [36].

### 2.4. Volatile Compounds

Untargeted volatile analysis of CS was performed using the headspace solid-phase micro-extraction technique (HS-SPME) and gas chromatography–quadrupole mass spectrometry (GC-*q*MS), according to a previously described method on coffee matrices [37]. The SPME fibre, a Carboxen/Polydimethylsiloxane/Divinylbenzene (CAR/PDMS/DVB), 2 cm × 50/30 μm film thickness (Supelco, Bellafonte, PA, USA), was exposed to the headspace of the sample using an SPME autosampler (PAL System, Combi PAL, Zwingen, Switzerland). Two millilitres of ultrapure water, 50 mg of CS, and 5 μL of α-thujone (internal standard, 5.15 mg/L) were placed in a 20 mL screw-cap glass vial fitted with a silicone–PTFE septum (Supelco, Milan, Italy) [38]. The vials were stirred at 10× *g* and 30 °C for 5 min, then the fibre was exposed to the headspace for 20 min at 30 °C. The fibre was then removed and inserted into the injector in splitless mode at 260 °C for 5 min. GC–*q*MS analysis was performed using a Shimadzu GC-2010 gas chromatograph equipped with a Shimadzu QP-2010 Plus quadrupole mass spectrometer (Shimadzu Corporation, Kyoto, Japan) and a DB-WAXETR capillary column (30 m × 0.25 mm, 0.25 μm film thickness, J&W Scientific Inc., Folsom, CA, USA). The temperature programme started at 40 °C and was maintained for 1 min, then increased at a rate of 3 °C/min to 200 °C, and finally increased at a rate of 10 °C/min to 250 °C (held for 5 min). The flow rate of the carrier gas (He) was 1 mL/min. The injection port temperature was 260 °C, and the ion source temperature and the interface temperatures were 250 °C. Detection was performed using electron ionisation at 70 eV with a scan range of 30–330 *m*/*z*. Tentative identification was performed using mass spectral matching against the NIST 05 MS library, combined with linear temperature-programmed retention indices. Analyte identification was confirmed using analytical standards, when available. The semi-quantitative concentrations of each volatile compound detected were calculated as the area of each volatile marker ion quantifier divided by the response factor of the internal standard using the ion quantifier (*m*/*z* 81) of the internal standard α-thujone as micrograms of α-thujone equivalents per kilogram of CS sample. All experiments were performed in triplicate.

### 2.5. Polyphenol Extraction

To extract the bioactive phenolics, a solvent mixture of 60% ethanol in ultrapure water was added to CS powder (sample-to-solvent ratio of 1:10 *w*/*v*). The mixture was shaken (100 oscillations/min) on an orbital shaker (VDRL 711, Asal s.r.l., Milan, Italy) at room temperature in the dark for 30 min. The extract was centrifuged (15 min, 4 °C, 16,800× *g*) and the supernatant was collected, filtered (PTFE 0.45 µm), and brought to volume (25 mL) with the extraction solvent [39]. Each sample was extracted in triplicate and immediately analysed.

### 2.6. HPLC-DAD-MS/MS Analysis

A Thermo-Finnigan SpectraSystem HPLC (Thermo-Finnigan, Waltham, MA, USA) equipped with a P2000 binary gradient pump, an SCM 1000 degasser, an AS 3000 automatic injector, and a Finnigan Surveyor PDA Plus detector, coupled in tandem with an API 3200 QTRAP with a Turbo V source (Applied Biosystems Sciex, Foster City, CA, USA), was used. Separation was achieved on a Kinetex 5 μm Phenyl-Hexyl 100 Å 150 × 4.6 mm (Phenomenex, Castel Maggiore, Italy) equipped with a SecurityGuard™ analytical guard cartridge system (Phenomenex). The mobile phase was composed of solvent A (formic acid 0.1% in ultrapure water) and solvent B (methanol) at a flow rate of 1 mL/min with injection volume of 10 µL. The elution programme was as follows: A 90% kept in isocratic for 0.5 min, A 70% in 2.5 min, A 65% in 5 min, A 60% in 3 min, A 20% in 19 min, and A 90% in 1 min maintained in isocratic for 1 min. Photodiode array (PDA) spectra were recorded in a full scan modality over the wavelength (λ) range of 220–600 nm, with quantification performed using a calibration curve obtained for the trans-5-CQA analytical standard. MS/MS conditions for the identification of caffeoylquinic acid (CQAs) were optimised using the *trans*-5-CQA standard. The ion source was operated in a negative ion mode using the following conditions: ion spray voltage, −4500 V; turbo spray temperature, 500 °C; curtain gas, 2.07 × 10^5^ Pa; interface heater, on; nebuliser gas, 2.4 × 10^5^ Pa; and heater gas, 10 × 10^5^ Pa. Nitrogen was used as the nebuliser, heater, curtain, and collision gas. Masses were recorded in the range of *m*/*z* 100–700 amu using an enhanced mass spectrum scan experiment with a declustering potential of −20 V and an entrance potential of −10 V. Product ions (MS/MS) were generated according to the information-dependent acquisition mode, with a threshold of 50,000 cps and a collision energy of −30 eV, and were collected in enhanced product ion mode.

### 2.7. Total Phenolic Content (TPC)

The TPC was spectrophotometrically assayed using the modified Folin–Ciocalteu’s method [40,41], as described by Belviso et al. [39]. The results were expressed as milligrams of gallic acid equivalents (GAE) per gram of dry powder based on a calibration curve of gallic acid (0–250 mg/L; R^2^ = 0.9987). All determinations were performed in triplicate.

### 2.8. Antioxidant Capacity

The radical scavenging capacity (RSA) of the extracts was assessed using a PerkinElmer 2030 Multilabel Reader with 96-well black microplates, as reported by Belviso et al. [39], according to the method described by von Gadow, Joubert, and Hansmann [42]. The results were expressed as μM of Trolox equivalents (TE) per gram of sample by means of a dose–response curve for Trolox (0–350 µM; R^2^ = 0.9986). Each sample was analysed in triplicate.

The oxygen radical absorbance capacity (ORAC) of the extracts was measured using a PerkinElmer 2030 Multilabel Reader with 96-well black microplates, as described by Belviso et al. [39]. Different Trolox solutions (0.25–6 µM) were used as standards to express the results as μM of TE per gram of sample. Analyses were performed in triplicate for each sample.

### 2.9. Statistical Analysis

Statistical analyses were performed using the STATISTICA software for Windows (Release 7.0; StatSoft Inc., Tulsa, OK, USA). One-way analysis of variance (ANOVA) with Tukey’s test for mean comparison was performed to highlight significant differences among the CSs. Factorial ANOVA using species (*Arabica* and *Canephora*), post-harvest treatment (wet and natural), and roasting intensity (light, medium, and dark) as factor production was applied.

## 3. Results

### 3.1. Dietary Fibre Profile

The soluble dietary fibre (SDF) and insoluble dietary fibre (IDF) contents of CSs are summarised in Table 1.

*Arabica* CS generally showed higher SDF content than that of the *Canephora* type, with this difference particularly evident in the washed samples. The highest content was found in the light-roasted washed *Arabica* CS and in the light-roasted natural *Canephora* CS. Within each subgroup, the differences between roasting conditions were low. Additionally, IDF content was not found to be significantly different between CSs based on coffee species, treatments, or roasting. The detected CS fibre content was in accordance with that reported in the literature [6,15,43]. Recently, Gottstein et al. [44] reported the DF content of both *Arabica* and *Canephora* CSs, which was comparable to our findings (62.0 ± 0.4 g/100 g for *Canephora* CS and 67.0 ± 1.0 g/100 g for *Arabica* CS). The abundance of DF (50–60%), of which approximately 85% is IDF, characterises this by-product to a greater extent than other dietary plant-based foods [10]. The high level of DF detected in the CS samples corroborates the hypothesis that CS has great potential as an ingredient in functional foods.

Factorial ANOVA (Table 2) confirmed that for IDF, only for the treatment, there was a significant difference in the highest concentration for the washed coffee. There were significant differences in the SDF between the species (*Arabica* showed higher amounts than *Canephora*) and roasting (light roast showed higher amounts than medium and dark).

### 3.2. Volatile Metabolites

Fifty-nine volatile compounds were found in CS samples (Table 3).

Among the volatile compounds, seven furan derivatives (furfural, 5-methyl-furfural, furfuryl formate, 2-acetyl furan, methyl 2-furoate, 2-acetyl-5-methyl-furan, and 2-furanmethanol), four terpenoids (linalool and linalool oxides), two alkyl pyrazine derivatives (methyl- and ethyl-pyrazine), three alcohols (phenyl ethyl alcohol, 2-heptanol, and 1-hexanol), and an indole derivative characterised natural *Arabica* CS, in contrast to washed *Arabica* CS samples or *Canephora* CS, independent of the roasting treatment. Thus, natural and washed *Canephora* CS and washed *Arabica* CS were poorly represented by all these compounds present at low concentrations. Findings on furans showed that 5-methyl-furfural may differentiate *Arabica* from *Canephora* CS species and separate the post-harvest treatments, becoming a possible CS post-harvest technological marker owing to its higher content in natural *Arabica* CS than in washed *Arabica* or *Canephora* CSs.

Conversely, in natural *Canephora* CS samples, higher amounts of alkyl pyrroles and sulphur compounds (dimethyl di- and tri-sulphide and thiophene) were detected, in contrast to the natural *Arabica* CS. Accordingly to Baggentoss et al. [31], sulphur molecules appeared to increase in coffee during overroasting. However, the 2-furfurylthiol, found in the *Arabica* coffee after the low-temperature roasting process [31], was not detected in the CS samples. Two di-sostituite alchyl pyrazines (2,5- and 2,6-dimethyl-pyrazine) were abundant in washed *Canephora* CS. The aroma of natural *Canephora* CS was characterised by higher amounts of pyridines and a phenolic compound with a spicy odour (4-vinyl guaiacol) derived from chlorogenic acid (CGA), as reported in *Canephora* [2,29] and *Arabica* [45] coffee.

Heterocyclic azotate compounds from the degradation of trigonelline (pyridine and N-methylpyrroles) were also present in the volatile fraction of the CSs. These compounds are produced in a model reaction system of the classic Maillard reaction. Pyridines are more abundant in natural *Canephora* CS, and are responsible for bitter, astringent, roasted, and burnt notes [46,47]. Finally, a high quantity of 2-furanmethanol, a volatile antioxidant heterocyclic compound [48], was detected in natural *Arabica* CS.

To better highlight the differences between samples, the volatile compounds were clustered into 10 chemical classes (Table 4).

As acetic acid did not show significant differences between the samples, this metabolite, associated with lactic acid bacterial metabolism [49], was not included in the volatile chemical classes of CSs.

Quantitatively, the major chemical class present in all CS samples was furan derivatives (range 5815–23,431 μg/kg), exhibiting malty and sweet roasted aromas derived mainly from the degradation of carbohydrates [47]. A significantly higher content of furans was found in light-roasted natural *Arabica* CS than in all other CSs, with 2-pentyl furan, furfural, and 5-methyl-furfural being the most abundant. 2-Pentyl furan, a low-quality indicator in black defective coffee seeds [47], was rarely detected in the natural *Canephora* CS. A heterocyclic component, 2-furanmethanol, which is formed by the degradation of sugars and quinic acid at high temperatures and is known to produce the bitter taste of roasted coffee [50], was mainly present in natural *Arabica* CS samples after a higher temperature roasting treatment. Consequently, this bitter volatile marker seemed to be more susceptible to dry treatment and a high roasting temperature. The second-most detectable group of volatiles in the CS samples was the pyrrole group (2425–10,000 μg/kg), particularly in natural *Canephora*, mainly as a result of dark roasting. Pyrroles are nitrogen-containing heterocycles produced by reactions between oxygen- and nitrogen-containing fragments [51] and antioxidant molecules formed through the Maillard reaction [52], with a peculiar sweet smell [47] and roasted or toasted flavours [32]. 1-Methylpyrrole, considered an off-flavour isolated from defective *Arabica* roasted coffee [18], was the most detectable natural *Canephora* dark-roasted CS.

Pyrazines, the second-most abundant odour class in roasted coffee powder [53], were poorly detected in CS samples, in the range of 268–746 μg/kg. Natural *Arabica* CS showed the highest content, whereas the lowest was in the washed *Arabica* CS. An inverse trend with respect to the coffee post-harvest treatment was exhibited by *Canephora* CS, which was characterised by a lower total content than that of *Arabica* CS. Methyl- and ethyl-pyrazines were more abundant in natural *Arabica*-roasted light CS (Table 3), with minimal detection in roasted washed *Arabica* and processed *Canephora* CSs.

Sulphur compounds exhibited significantly higher content in natural *Canephora* dark-roasted CS compared to other CS samples and a lower content in washed CS of both species except for dark-roasted washed Arabica (1735 ± 65 μg/kg), which presents higher content than dark-roasted natural Arabica (1470.27 ± 118.9 μg/kg).

Factorial ANOVA (Table 5) highlighted that coffee species had a significant effect on all classes of volatile compounds. Only pyrazines had no significant differences between the species. In addition, for the cherry treatments (natural and washed), all classes of volatile compounds showed significant differences. In particular, the CSs detected after natural treatment showed the highest values. There were significant differences in roasting intensity only for sulphur compounds (higher amounts for dark roasting) and terpenoids (higher amounts for light roasting). Consequently, the interaction between the species and roasting treatment was not significant.

### 3.3. Phenolic Compounds

The HPLC-MS/MS analysis of the CS extracts highlighted the presence of seven compounds belonging to CGAs (Table 6), a family of natural hydroxycinnamic acid esters, displaying antimicrobial, antifungal [54], and oxidative and inflammatory stress control activity [55]. Of these, three were isomers of caffeoylquinic acid, namely 3-*O*-caffeoylquinic (3-*O*-CQA), 5-*O*-caffeoylquinic (5-*O*-CQA), and 4-*O*-caffeoylquinic acid (4-*O*-CQA); two were isomers of feruloylquinic acids (FQAs); and two were isomers of dicaffeoylquinic acids (di-CQA) (Table 6). 5-*O*-CQA was identified by comparison with analytical standards, whereas 3-*O*-CQA, 4-*O*-CQA, FQAs, and di-CQAs were identified based on data reported in the literature [56,57]. Typically, CGAs are abundant in coffee, in different concentrations and isomeric mixtures, and determine the phenolic composition of CS. Nzekoue et al. [13] analysed different C. *Arabica* extracts and reported the presence of only three caffeoylquinic acids (3-CQA, 5-CQA, and 3,5-diCQA); however, Bresciani et al. [14] detected nine different CGAs, including caffeoylquinic lactones and coumaroylquinic acids. In general, caffeoylquinic acid isomers are the most abundant group [14,43].

In our CS extracts, 5-*O*-CQA was the most abundant CGA acid, whereas 3-O-CQA was detected only in dark- and medium-roasted washed *Arabica* CS (Table 7).

The 5-*O*-CQA content was in the range of 0.41–2.96 mg/g, with the maximum amount in the dark-roasted washed *Arabica* CS. On the contrary, *Canephora* CS is characterised by the lowest 5-*O*-CQA amount independently to roasting intensity or post-harvest treatment. The content of 4-*O*-CQA varied from a minimum of 0.08 mg/g in washed *Canephora* CS obtained from a medium or dark roasting to a maximum of 0.50 mg/g in washed *Arabica* CS obtained with a dark roasting. Again, a significant effect of the roasting degree was detected only in washed *Arabica* CS, with an increase in 4-*O*-CQA with the level of roasting. This trend was unexpected because, as the degree of roasting increases, caffeoylquinic acids were generally thermally degraded to their corresponding lactones [55]. However, many factors are involved in the roasting process as the roasting profile (time and temperature conditions), together with the type of roaster and the roasting speed, can affect the CGAs’ content. For the same roasting degree, the higher the roasting speed, the lower the CGAs’ loss [58]. In the same samples, as the degree of roasting increased, a decrease in SDF was observed, likely leading to a lower formation of fibre–antioxidant complexes.

Among FQAs, FQA isomer 2 was the most abundant, with the highest content found both in washed *Arabica* and natural *Canephora* CS obtained with a dark roasting (0.30 mg/g), while the lowest content in natural *Arabica* CS was produced with medium roasting (0.10 mg/g). On average, these values were similar to those reported in the literature [14]. The FQA1 content ranged from 0.04 mg/g in natural *Canephora* to 0.10 mg/g in washed *Canephora* CS, whereas the content of this compound was the same in all *Arabica* CSs. The amount of the two di-CQAs was higher in the dark-roasted washed *Arabica* CS, whereas the lowest content was detected in natural *Arabica* CS subjected to medium roasting. The content range was 0.14–0.72 mg/g for di-CQA1 and 0.08–0.45 mg/g for di-CQA2. Of the *Canephora* CSs, the highest values of di-CQAs were found in medium-roasted washed *Canephora* CS, with 0.33 mg/g and 0.41 mg/g for di-CQA 1 and di-CQA 2, respectively.

These data, which are difficult to compare quantitatively with those reported in the literature due to differences in the extraction techniques or expression of analytical data, are consistent from a qualitative point of view, with caffeoylquinic acids the main component of the phenolic fraction and minor CGAs represented by feruloylquinc acids. It has been reported that all factors that characterise green coffee beans during roasting affect the CGAs’ isomer composition [55]. Regarding the coffee variety, green *Canephora* beans contained a higher content of CGAs compared to *Arabica* beans, with a similar trend in their respective CSs [59]. Contrary to what has been reported, the CS analysed in this study showed the opposite trend, which was particularly evident in the washed samples. These differences may be explained by the geographical origin of the species and the type of post-harvest treatment used, which can play an equally important role in defining the chemical composition of coffee beans [5].

Factorial ANOVA (Table 8) shows that all the production factors (species, treatment, and roasting) and all the interactions significantly affected the content of all phenolic compounds detected in the CS samples.

### 3.4. Total Phenolic Content and Antioxidant Capacity

Table 9 shows the mean values of TPC and the antioxidant capacity of the CS extracts.

Natural *Arabica* CS had a significantly higher TPC content. Overall, samples subjected to natural post-harvest treatment had higher TPCs, similar to those reported in a previous study [21], with *Arabica* CSs generally characterised by higher TPCs than those of *Canephora*. This trend contrasts that reported by other authors [59] but followed the tendency observed with respect to the content of phenolic compounds.

Significantly higher DPPH and ORAC values (average of 50 μmol TE/g and 348 μmol TE/g, respectively) were detected in natural *Arabica* CS, independent of the roasting process. However, it was difficult to compare our results with those reported in the literature because of different solvent extraction procedures and type of expression of results, often attributed to the extract rather than the sample.

With respect to the roasting process, the TPC, DPPH, and ORAC assays deferred. In most cases, we observed an increase in TPC values with an increase in the roasting degree (above all for washed *Arabica*), whereas DPPH and ORAC values were characterised by more variable trends, such as that ORAC increased or decreased alternatively with the roasting degree. The different reaction mechanisms and/or affinities for specific antioxidants in the adopted assays can be linked to the balance between the degradation rate of phenolic compounds and the generation of the Maillard reaction products that characterise the roasting process. It is well known that during roasting, the majority of phenolics present in coffee beans are partially destroyed and/or bound to high-molecular-weight polymers, mainly melanoidins, which are responsible for the strong antioxidant properties and metal-chelating ability of coffee brews [60], in addition to diterpenes that are present in CS and possess relevant antioxidant activities [13]. Nevertheless, Nicoli et al. [61] reported a progressive reduction in the antioxidant capacity of coffee brews with the degree of roasting, with the highest capacity in medium-roasted coffee. As reported by Komes and Bušić [60], high-molecular-weight polymerised melanoidins contribute to the coffee brew antioxidant capacity less than low-molecular-weight Maillard reaction products.

Factorial ANOVA (Table 10) highlighted that the treatment was significant for all parameters evaluated, with the highest values for natural products. Coffee species was significant only for TPC and RSA, whereas roasting was significant only for TPC, with the highest values for the dark-roasted product.

All interactions showed significant effects on TPC, whereas for the antioxidant parameters, only some interactions showed significant effects.

## 4. Conclusions

The present study assessed the effects of botanical origin and technological parameters (post-harvest treatment, drying, and roasting) on the chemical composition of CS. Natural *Arabica* CS, with a higher content of volatile compounds (Maillard and varietal origins), TPC, and antioxidant capacity, is significantly different to washed *Arabica* CS, highlighting the effect of post-harvest coffee bean treatments on CS composition. Additionally, washed *Arabica* CS showed greater preservation of soluble dietary fibre and chlorogenic derivatives. *Canephora* CS revealed differences in volatile compounds from the *Arabica* species owing to the higher content of pyrroles, sulphur compounds, and pyridines. No clear trend was observed for the effect of the roasting degree. Therefore, different coffee attributes seem to be reflected in the chemical properties of this coffee by-product, with further research required to elucidate and potentiate these features.

## Figures and Tables

**Table 1:** Content (%; mean ± standard deviation) of dietary fibre in CS samples based on species, post-harvest treatments, and roasting intensity, and results of ANOVA with Tukey’s test for mean comparison.

			SDF	IDF
Natural	*Canephora*	Light	10.2 ± 0.90 bcde	49.1 ± 2.70
		Medium	7.8 ± 0.80 abc	46.7 ± 2.49
		Dark	7.6 ± 0.80 ab	47.2 ± 2.49
	*Arabica*	Light	11.7 ± 1.10 ef	47.0 ± 2.50
		Medium	10.9 ± 0.99 def	47.9 ± 2.60
		Dark	12.6 ± 1.10 ef	47.4 ± 2.60
Washed	*Canephora*	Light	8.0 ± 0.80 abc	49.1 ± 2.70
		Medium	5.7 ± 0.70 a	50.1 ± 2.20
		Dark	8.4 ± 0.89 abcd	48.1 ± 2.59
	*Arabica*	Light	13.4 ± 1.20 f	51.2 ± 2.19
		Medium	11.7 ± 1.10 ef	50.6 ± 2.20
		Dark	10.5 ± 0.90 cde	51.6 ± 2.20
Significance			***	ns

Different letters within a column indicate significant differences according to Tukey’s test (*** *p* < 0.001; ns: not significant). SDF: soluble dietary fibre; IDF: insoluble dietary fibre.

**Table 2:** Values of significance determined with factorial ANOVA performed on production factors and their interactions for SDF and IDF values.

	Species (S)	Treatment (T)	Roasting (R)	S × T	S × R	T × R	S × T × R
**SDF**	0.000	0.117	0.000	0.052	0.313	0.840	0.000
**IDF**	0.284	0.005	0.873	0.181	0.660	0.895	0.444

**Table 3:** Content (μg/kg; mean ± standard deviation) of volatile compounds identified in CS samples.

	**Washed**					
	***Canephora***			***Arabica***		
	**Light**	**Medium**	**Dark**	**Light**	**Medium**	**Dark**
**Pyrroles**						
1-methyl-pyrrole	1251.90 ± 78.49	1609.56 ± 51.72	1067.36 ± 50.44	1394.81 ± 198.7	1460.39 ± 133.85	1963.01 ± 49.34
1-ethyl-pyrrole	694.41 ± 4.92	474.82 ± 8.53	356.15 ± 46.49	246.55 ± 46.62	292.73 ± 22.86	259.27 ± 109.96
1-pentyl-pyrrole	981.21 ± 41.82	636.35 ± 55.84	562.95 ± 65.38	160.55 ± 24.53	237.19 ± 44.92	214.84 ± 3.57
pyrrole	533.38 ± 5.32	504.53 ± 28.89	326.06 ± 39.78	284.17 ± 62.43	316.42 ± 46.40	509.91 ± 25.64
1-methyl-pyrrole-2-carboxaldehyde	77.64 ± 5.93	58.14 ± 7.19	61.60 ± 8.07	87.50 ± 25.05	107.72 ± 14.97	115.49 ± 9.23
2-acetyl-1-methyl-pyrrole	16.56 ± 0.80	10.61 ± 1.62	12.34 ± 2.10	13.80 ± 2.67	16.15 ± 2.25	16.94 ± 0.90
4-ethyl-2-methyl-pyrrole	47.05 ± 2.05	47.31 ± 2.77	43.12 ± 3.93	11.28 ± 3.06	11.40 ± 1.30	16.08 ± 0.62
1-furfuryl-pyrrole	214.71 ± 2.96	124.27 ± 9.91	121.91 ± 10.93	100.20 ± 11.79	115.61 ± 11.36	132.21 ± 2.79
2-acetyl-pyrrole	59.22 ± 14.65	22.24 ± 5.27	34.25 ± 7.63	22.78 ± 2.52	45.84 ± 11.90	28.99 ± 2.25
1H-pyrrole-2-carboxaldehyde	170.20 ± 54.82	64.28 ± 15.64	73.27 ± 3.71	104.26 ± 16.33	194.13 ± 54.17	118.18 ± 12.37
**Pyrazines**						
2-methyl-pyrazine	217.09 ± 2.03	244.91 ± 50.53	251.04 ± 49.33	163.91 ± 60.36	136.38 ± 37.05	170.44 ± 15.83
2,6-dimethyl-pyrazine	58.03 ± 0.71	75.32 ± 19.81	71.78 ± 10.42	36.24 ± 15.92	27.88 ± 10.10	29.34 ± 3.77
2,5-dimethyl-pyrazine	54.93 ± 1.72	65.24 ± 9.32	59.49 ± 10.60	36.23 ± 15.36	26.57 ± 9.92	32.08 ± 4.02
2-ethyl-pyrazine	116.96 ± 0.64	123.86 ± 18.15	112.57 ± 15.10	76.22 ± 24.95	77.45 ± 19.21	92.60 ± 4.30
**Furan derivatives**						
2-pentyl-furan	4625.53 ± 227.61	6613.43 ± 222.83	6125.76 ± 771.02	4307.49 ± 784.14	3226.46 ± 1475.85	5674.83 ± 151.70
furfural	1305.24 ± 28.81	1110.35 ± 109.00	1209.18 ± 157.76	2072.72 ± 543.82	2013.86 ± 303.80	2720.21 ± 156.88
furfuryl formate	28.89 ± 4.32	33.71 ± 4.26	26.78 ± 2.85	28.92 ± 6.76	20.35 ± 2.98	33.38 ± 5.17
2-acetyl furan	81.07 ± 2.79	59.34 ± 7.50	63.33 ± 8.94	68.40 ± 24.74	84.94 ± 15.20	100.21 ± 12.57
furfurylacetate	85.13 ± 0.55	117.89 ± 7.47	76.75 ± 5.65	82.32 ± 21.51	65.05 ± 10.65	81.50 ± 4.98
5-methyl-furfural	405.83 ± 11.21	359.22 ± 60.48	426.01 ± 51.36	458.08 ± 124.92	556.82 ± 88.51	620.23 ± 33.97
methyl 2-furoate	175.49 ± 0.14	123.45 ± 15.17	132.78 ± 14.94	175.06 ± 37.27	206.17 ± 25.81	237.94 ± 10.01
2-methyl-benzofuran	34.32 ± 2.02	27.33 ± 3.24	27.90 ± 4.05	23.56 ± 2.27	31.96 ± 4.85	28.21 ± 7.01
2-acetyl-5-methyl-furan	11.42 ± 1.00	8.71 ± 1.41	12.66 ± 1.21	31.04 ± 7.74	32.61 ± 5.32	36.02 ± 4.82
2-furanmethanol	323.02 ± 26.34	265.50 ± 11.74	427.83 ± 88.07	222.28 ± 91.44	380.82 ± 49.71	394.50 ± 9.55
**Pyridines**						
pyridine	453.61 ± 50.95	455.99 ± 72.79	374.43 ± 53.72	155.63 ± 36.84	237.41 ± 43.93	268.44 ± 13.63
2-acetylpyridine	1343.26 ± 27.22	914.10 ± 54.48	801.57 ± 85.20	288.40 ± 78.89	390.00 ± 48.87	367.76 ± 15.52
**Sulphureous compounds**					
thiophene	467.34 ± 3.65	366.51 ± 24.43	272.60 ± 21.98	244.57 ± 40.85	274.90 ± 22.75	358.48 ± 12.98
dimethyl disulphide	1037.48 ± 94.03	963.95 ± 40.66	675.75 ± 54.18	701.26 ± 19.40	864.42 ± 121.34	1233.14 ± 47.69
dimethyl trisulphide	116.41 ± 7.59	120.95 ± 17.13	112.38 ± 14.63	73.11 ± 21.75	104.50 ± 15.99	144.12 ± 29.00
**Terpenoids**						
limonene	29.10 ± 3.92	43.37 ± 26.12	26.26 ± 9.67	43.39 ± 7.59	34.26 ± 7.33	36.76 ± 13.84
*cis* dehydroxy linalool oxide	ND	ND	ND	201.21 ± 4.86	171.26 ± 34.13	154.70 ± 26.02
*trans* dehydroxy linalool oxide	ND	ND	ND	48.62 ± 2.27	32.30 ± 15.09	47.68 ± 4.88
*cis* furanoid linalool oxide	9.69 ± 1 .28	6.01 ± 1.05	8.27 ± 0.68	54.48 ± 10.39	57.03 ± 14.82	48.92 ± 1.63
linalool	ND	ND	ND	10.47 ± 1.54	13.10 ± 1.32	14.25 ± 1.30
𝛼-ionone	14.29 ± 2.74	12.24 ± 0.93	13.39 ± 0.84	7.12 ± 1.16	8.83 ± 1.66	9.48 ± 3.09
**Acids**						
acetic acid	681.32 ± 178.86	291.62 ± 148.52	450.89 ± 325.72	280.69 ± 99.05	329.11 ± 170.02	386.55 ± 174.63
**Aromatic compounds**					
toluene	2769.40 ± 82.64	2400.55 ± 40.08	2346.66 ± 190.27	2419.83 ± 371.11	2785.85 ± 308.59	3735.39 ± 396.59
styrene	94.56 ± 8.54	131.29 ± 43.84	156.31 ± 83.14	55.18 ± 31.61	91.82 ± 12.97	173.67 ± 50.08
trimethylbenzene	327.29 ± 4.20	293.26 ± 10.45	274.53 ± 25.99	227.20 ± 24.65	231.01 ± 31.13	401.85 ± 56.17
acetophenone	54.88 ± 4.17	33.05 ± 0.48	40.59 ± 3.97	33.93 ± 5.50	41.19 ± 8.58	38.57 ± 7.76
1,2-dihydro-1,5,8-trimethyl-naphthalene ^§^	43.66 ± 1.72	33.57 ± 2.15	33.25 ± 2.89	17.34 ± 2.47	19.73 ± 2.70	17.46 ± 2.42
phenyl ethyl formate	26.72 ± 1.38	19.38 ± 1.97	22.49 ± 2.70	22.02 ± 2.75	23.48 ± 2.15	26.00 ± 0.25
2-methoxy-phenol	70.40 ± 14.70	28.13 ± 4.82	40.98 ± 12.43	12.96 ± 1.47	45.23 ± 6.87	24.43 ± 1.77
phenol	98.73 ± 19.95	58.62 ± 8.91	86.60 ± 22.63	40.22 ± 10.40	65.58 ± 18.11	58.59 ± 14.50
4-vinylguaiacol	44.33 ± 12.48	18.74 ± 4.75	25.25 ± 12.50	10.49 ± 0.39	23.94 ± 8.30	15.09 ± 1.11
4-methylacetophenone	65.09 ± 3.88	60.22 ± 5.03	68.05 ± 6.69	154.40 ± 16.41	167.63 ± 16.24	177.59 ± 7.09
**Ketones**						
6-methyl-5-hepten-2-one	26.19 ± 0.26	20.90 ± 6.05	14.96 ± 2.41	56.57 ± 11.00	51.00 ± 0.63	63.91 ± 4.52
2-nonanone	79.56 ± 0.75	73.32 ± 8.91	61.30 ± 9.67	76.00 ± 13.37	74.50 ± 12.20	86.67 ± 2.88
2-decanone	49.69 ± 3.03	50.28 ± 1.53	47.93 ± 2.90	58.50 ± 10.37	58.94 ± 3.45	59.52 ± 2.97
**Aldehydes**						
hexanal	1698.95 ± 74.68	1435.68 ± 142.83	1135.59 ± 144.34	1973.71 ± 191.22	1687.14 ± 256.23	2686.55 ± 4.86
heptanal	268.28 ± 8.32	290.11 ± 40.88	258.76 ± 20.81	411.78 ± 73.93	383.99 ± 13.47	499.33 ± 15.02
benzaldehyde	1240.27 ± 8.67	890.76 ± 78.48	964.53 ± 96.91	783.80 ± 136.48	849.38 ± 66.89	1021.72 ± 31.37
3-methyl-benzaldehyde	57.35 ± 1.35	57.33 ± 6.45	62.61 ± 6.45	159.92 ± 21.93	154.37 ± 9.77	184.13 ± 12.71
benzeneacetaldehyde	548.47 ± 27.30	442.92 ± 63.32	462.78 ± 35.86	205.28 ± 23.60	245.23 ± 29.04	260.02 ± 9.75
**Alcohols**						
2-heptanol	268.09 ± 9.47	213.53 ± 14.07	224.82 ± 27.28	194.62 ± 50.13	214.99 ± 24.08	216.78 ± 11.19
1-hexanol	85.86 ± 0.14	74.36 ± 6.35	73.64 ± 8.42	44.15 ± 7.48	52.99 ± 10.39	69.82 ± 9.25
2-butoxy-ethanol	277.55 ± 40.67	269.09 ± 10.26	153.40 ± 53.11	158.67 ± 14.27	284.52 ± 70.93	381.25 ± 78.67
phenylethyl alcohol	201.44 ± 58.12	77.54 ± 14.40	122.98 ± 62.17	115.91 ± 12.54	165.86 ± 44.94	120.76 ± 5.93
**Indole**						
5-hydroxy-1H-indole ^§^	29.68 ± 0.82	15.54 ± 1.85	14.52 ± 2.43	14.54 ± 2.21	15.13 ± 1.56	17.45 ± 1.29
	**Natural**					
	***Canephora***			***Arabica***		
	**Light**	**Medium**	**Dark**	**Light**	**Medium**	**Dark**
**Pyrroles**						
1-methyl-pyrrole	2650.90 ± 411.16	2702.50 ± 577.42	3540.61 ± 258.43	2465.86 ± 154.31	1827.32 ± 103.42	1687.60 ± 162.71
1-ethyl-pyrrole	1159.20 ± 190.77	1217.46 ± 299.55	1552.12 ± 106.62	762.60 ± 36.82	602.27 ± 36.29	518.20 ± 13.33
1-pentyl-pyrrole	1652.01 ± 322.26	1509.49 ± 346.66	2203.74 ± 132.42	365.84 ± 28.24	353.81 ± 19.15	296.92 ± 2.97
pyrrole	1450.12 ± 235.54	1922.16 ± 533.99	1916.90 ± 298.47	689.08 ± 46.17	577.45 ± 31.28	529.06 ± 25.53
1-methyl-pyrrole-2-carboxaldehyde	95.75 ± 16.14	109.73 ± 30.49	129.25 ± 32.00	119.84 ± 13.66	108.43 ± 1.67	92.90 ± 9.69
2-acetyl-1-methyl-pyrrole	18.20 ± 2.34	22.20 ± 7.17	29.63 ± 7.08	27.25 ± 3.49	24.31 ± 0.38	23.45 ± 1.82
4-ethyl-2-methyl-pyrrole	23.25 ± 5.21	19.29 ± 4.92	26.59 ± 4.46	24.60 ± 1.59	24.35 ± 1.37	20.78 ± 1.21
1-furfuryl-pyrrole	336.60 ± 58.21	335.34 ± 81.72	464.59 ± 58.23	641.89 ± 31.71	563.98 ± 25.47	494.38 ± 14.98
2-acetyl-pyrrole	51.40 ± 11.36	59.37 ± 54.33	40.18 ± 13.81	59.41 ± 4.16	70.72 ± 5.82	63.55 ± 4.06
1H-pyrrole-2-carboxaldehyde	86.26 ± 17.75	164.47 ± 155.16	96.95 ± 33.64	133.26 ± 9.82	160.39 ± 13.64	139.25 ± 13.08
**Pyrazines**						
2-methyl-pyrazine	212.05 ± 41.88	189.89 ± 46.56	235.22 ± 91.88	413.01 ± 59.97	390.79 ± 68.81	329.02 ± 12.63
2,6-dimethyl-pyrazine	45.71 ± 7.47	42.93 ± 13.40	54.50 ± 18.87	80.40 ± 14.36	73.87 ± 6.63	59.97 ± 6.44
2,5-dimethyl-pyrazine	41.16 ± 7.58	37.54 ± 10.57	45.26 ± 14.50	65.88 ± 7.63	67.04 ± 3.85	51.60 ± 8.24
2-ethyl-pyrazine	130.78 ± 19.91	126.25 ± 39.54	152.10 ± 63.07	187.12 ± 15.63	184.26 ± 9.13	161.47 ± 10.68
Furan derivatives						
2-pentyl-furan	3837.49 ± 797.32	3268.96 ± 888.35	4607.14 ± 492.76	9202.51 ± 799.60	6736.12 ± 457.01	5659.82 ± 677.2
furfural	1196.27 ± 139.12	1360.04 ± 234.56	1530.50 ± 390.06	10,733.26 ± 862.80	9701.92 ± 486.65	7491.19 ± 491.95
**furfuryl formate**	30.72 ± 4.01	25.49 ± 4.92	53.54 ± 7.67	101.87 ± 9.60	91.07 ± 0.46	81.34 ± 7.62
2-acetyl furan	48.15 ± 2.69	69.19 ± 15.94	84.22 ± 28.00	448.91 ± 24.72	417.32 ± 35.66	344.70 ± 35.03
furfurylacetate	60.86 ± 9.33	69.53 ± 12.50	117.96 ± 20.86	126.14 ± 12.66	118.87 ± 2.05	103.38 ± 4.87
5-methyl-furfural	335.22 ± 36.52	414.95 ± 101.62	405.37 ± 110.94	1761.17 ± 141.47	1664.01 ± 76.20	1445.55 ± 119.58
methyl 2-furoate	200.23 ± 26.29	254.46 ± 52.60	288.18 ± 68.80	431.98 ± 29.46	387.84 ± 9.96	334.91 ± 17.68
2-methyl-benzofuran	34.04 ± 6.88	38.07 ± 14.80	40.29 ± 5.46	65.51 ± 3.91	68.37 ± 1.86	58.79 ± 1.17
2-acetyl-5-methyl-furan	7.78 ± 1.37	10.61 ± 5.00	7.82 ± 2.83	50.04 ± 9.25	49.66 ± 5.29	38.21 ± 3.98
2-furanmethanol	208.95 ± 49.90	304.43 ± 217.36	289.85 ± 86.91	509.73 ± 60.05	595.15 ± 41.80	557.10 ± 95.58
**Pyridines**						
pyridine	351.95 ± 53.51	411.19 ± 73.97	579.64 ± 101.52	161.57 ± 16.00	104.05 ± 15.19	104.22 ± 19.20
2-acetylpyridine	2267.70 ± 394.32	2111.30 ± 463.02	2977.01 ± 146.19	775.45 ± 131.27	751.76 ± 42.90	646.48 ± 15.84
**Sulphureous compounds**						
thiophene	880.14 ± 113.05	883.42 ± 100.30	1366.88 ± 86.85	388.65 ± 11.34	401.47 ± 16.33	350.90 ± 9.92
dimethyl disulphide	1616.88 ± 251.81	1712.16 ± 409.26	2742.16 ± 103.16	1437.51 ± 111.31	1000.18 ± 68.21	968.48 ± 109.95
dimethyl trisulphide	219.93 ± 52.04	208.61 ± 92.86	403.39 ± 25.72	189.56 ± 26.05	131.39 ± 9.73	150.90 ± 21.06
**Terpenoids**						
limonene	52.77 ± 15.31	21.73 ± 12.43	53.09 ± 16.46	63.79 ± 9.66	79.78 ± 8.38	64.11 ± 10.28
*cis* dehydroxy linalool oxide	ND	ND	ND	862.32 ± 64.60	1210.55 ± 69.08	755.19 ± 182.87
*trans* dehydroxy linalool oxide	ND	ND	ND	445.90 ± 79.05	567.20 ± 34.36	211.31 ± 16.31
*cis* furanoid linalool oxide	9.98 ± 3.54	17.43 ± 11.09	7.09 ± 1.92	333.56 ± 35.09	327.59 ± 79.99	312.87 ± 13.06
linalool	5.44 ± 1.33	5.87 ± 1.55	4.21 ± 0.43	87.27 ± 5.74	75.71 ± 12.62	73.14 ± 1.60
𝛼-ionone	12.08 ± 1.26	9.23 ± 2.74	13.04 ± 1.97	12.38 ± 1.48	11.88 ± 1.50	10.76 ± 1.26
**Acids**						
acetic acid	362.42 ± 353.01	402.30 ± 298.82	216.77 ± 98.63	724.92 ± 306.15	674.44 ± 238.20	269.18 ± 57.53
**Aromatic compounds**						
toluene	3356.28 ± 1188.46	4134.86 ± 262.32	3840.50 ± 152.52	2591.01 ± 75.59	2688.70 ± 113.84	2273.74 ± 92.41
styrene	285.07 ± 70.58	317.78 ± 181.90	462.58 ± 202.47	108.97 ± 35.25	228.21 ± 44.85	164.03 ± 19.67
trimethylbenzene	206.98 ± 65.82	326.10 ± 70.82	390.79 ± 15.52	397.66 ± 110.41	270.70 ± 28.60	228.37 ± 14.14
acetophenone	57.63 ± 17.93	65.81 ± 8.54	58.81 ± 8.31	79.23 ± 53.48	47.96 ± 11.45	32.87 ± 0.98
1,2-dihydro-1,5,8-trimethyl-naphthalene ^§^	59.54 ± 11.77	54.99 ± 14.69	82.66 ± 2.05	32.09 ± 1.17	27.81 ± 1.92	23.25 ± 0.55
phenyl ethyl formate	27.88 ± 5.03	30.01 ± 10.71	33.27 ± 4.06	115.27 ± 3.38	101.29 ± 4.45	94.70 ± 3.14
2-methoxy-phenol	58.25 ± 10.97	116.90 ± 96.46	77.00 ± 9.93	27.46 ± 4.37	25.19 ± 0.56	24.02 ± 2.90
phenol	65.01 ± 27.93	114.91 ± 64.98	73.58 ± 15.56	94.63 ± 70.49	58.52 ± 13.34	47.76 ± 3.74
4-vinylguaiacol	55.46 ± 13.41	55.93 ± 46.42	69.32 ± 12.13	16.48 ± 2.92	16.40 ± 2.15	15.71 ± 0.77
4-methylacetophenone	93.72 ± 19.03	85.39 ± 32.03	101.91 ± 11.32	238.52 ± 8.67	217.37 ± 9.39	200.85 ± 8.87
**Ketones**						
6-methyl-5-hepten-2-one	38.59 ± 0.52	37.06 ± 18.25	42.89 ± 14.49	84.87 ± 5.89	77.76 ± 4.68	64.43 ± 1.65
2-nonanone	43.54 ± 18.28	44.56 ± 10.93	26.46 ± 23.56	67.72 ± 2.74	58.96 ± 5.60	45.09 ± 1.25
2-decanone	29.27 ± 5.64	27.91 ± 8.53	34.42 ± 1.74	42.07 ± 4.24	39.18 ± 2.69	28.39 ± 1.04
**Aldehydes**						
hexanal	1404.66 ± 157.54	1542.63 ± 415.42	1135.25 ± 42.38	2697.24 ± 134.52	2184.86 ± 194.80	2046.37 ± 93.57
heptanal	258.11 ± 18.91	220.06 ± 36.25	245.03 ± 14.95	390.72 ± 37.22	366.24 ± 25.48	286.95 ± 4.46
benzaldehyde	1478.76 ± 239.36	1245.71 ± 271.55	1750.03 ± 152.18	1218.69 ± 112.61	1127.80 ± 72.58	913.87 ± 27.75
3-methyl-benzaldehyde	40.35 ± 10.99	41.05 ± 16.33	35.95 ± 2.62	188.57 ± 2.12	175.46 ± 6.04	144.22 ± 2.58
benzeneacetaldehyde	405.63 ± 35.14	362.11 ± 146.94	449.94 ± 115.47	362.33 ± 37.43	377.68 ± 42.45	274.43 ± 23.11
**Alcohols**						
2-heptanol	179.24 ± 25.96	186.66 ± 50.79	220.39 ± 24.45	2433.20 ± 134.42	2237.48 ± 85.04	1737.06 ± 27.84
1-hexanol	40.60 ± 5.96	43.53 ± 5.64	46.54 ± 4.95	280.05 ± 31.09	254.36 ± 7.20	191.38 ± 2.61
2-butoxy-ethanol	289.55 ± 111.57	172.12 ± 114.19	95.21 ± 10.19	330.91 ± 357.52	207.12 ± 76.78	316.65 ± 150.32
phenylethyl alcohol	181.34 ± 70.42	226.23 ± 187.68	161.26 ± 45.50	480.88 ± 53.66	525.13 ± 42.63	468.86 ± 48.14
**Indole**						
5-hydroxy-1H-indole ^§^	42.80 ± 10.12	41.71 ± 9.24	56.07 ± 6.17	88.87 ± 0.39	87.76 ± 5.40	77.47 ± 0.76

^§^: Tentative identification performed only by mass spectral matching against NIST 05 MS library; ND—not detected.

**Table 4:** Content (μg/kg; mean ± standard deviation) of volatile classes detected in CS samples according to species, post-harvest treatment, and roasting intensity, and results of variance analysis with Tukey’s test for mean comparison.

Post-Harvest Treatment	Washed						Natural						Significance
Species	*Canephora*			*Arabica*			*Canephora*			*Arabica*			
Roasting Degree	Light	Medium	Dark	Light	Medium	Dark	Light	Medium	Dark	Light	Medium	Dark	
Pyrroles	4046 ± 199 ab	3552 ± 142 ab	2658 ± 213 a	2425 ± 371 a	2797 ± 214 a	3374 ± 118 ab	7523 ± 1156 c	8062 ± 2003 cd	10,000 ± 854 d	5289 ± 288 b	4313.03 ± 26.62 ab	3866.08 ± 124.56 ab	***
Pyrazines	447 ± 1 abc	509 ± 97 abcd	494 ± 84 abcd	312 ± 116 a	268 ± 75 a	324 ± 25 a	429 ± 74 a	396 ± 109 a	487 ± 187 abcd	746 ± 96 d	715.96 ± 80.78 cd	602.06 ± 36.12 bcd	***
Furans	7075 ± 294 ab	8718 ± 431 ab	8528 ± 988 ab	7469 ± 1453 ab	6619 ± 1037 a	9927 ± 376 b	5959 ± 979 a	5815 ± 1536 a	7424 ± 919 ab	23,431 ± 1411 e	19,830.33 ± 369.45 d	16,114.97 ± 1126.57 c	***
Pyridines	1796 ± 23 d	1370 ± 88 cd	1176 ± 121 bcd	444 ± 113 a	627 ± 37 ab	636 ± 29 ab	2619 ± 446 e	2522 ± 526 e	3556 ± 71 f	937 ± 135 abc	855.81 ± 57.89 abc	750.70 ± 9.77 abc	***
Sulphur compounds	1621 ± 97 abc	1451 ± 77 abc	1060 ± 85 ab	1018 ± 41 a	1243 ± 159 ab	1735 ± 65 bc	2716 ± 416 d	2804 ± 579 d	4512 ± 171 e	2015 ± 137 c	1533.04 ± 93.28 abc	1470.27 ± 118.92 abc	***
Aromatics	3595 ± 45 ab	3076 ± 20 a	3094 ± 290 a	2993 ± 395 a	3495 ± 376 ab	4668 ± 514 bcd	4265 ± 1292 abcd	5302 ± 780 d	5190 ± 293 cd	3701 ± 327 abc	3682.15 ± 160.52 abc	3105.29 ± 84.97 a	***
Ketones	155 ± 3 abcd	144 ± 12 abc	124 ± 13 ab	191 ± 34 cd	184 ± 14 bcd	210 ± 6 d	111 ± 23 a	109 ± 37 a	103 ± 39 a	194.65 ± 6 cd	175 ± 11 bcd	137 ± 1 abc	***
Terpenoids	53 ± 7 a	61 ± 28 a	47 ± 9 a	365 ± 63 c	316 ± 69 c	311 ± 16 bc	80 ± 12 ab	54 ± 8 a	77 ± 12 ab	1805.22 ± 38 e	2272 ± 177 f	1427 ± 181 d	***
Aldehydes	3813 ± 120 abcd	3116 ± 200 a	2884 ± 260 a	3534 ± 419 ab	3320 ± 366 ab	4651 ± 48 ab	3587 ± 446 ab	3411 ± 844 ab	3616 ± 307 abc	4857.54 ± 245 d	4232 ± 153 bcd	3665 ± 102 abc	***
Alcohols	832 ± 26 a	634 ± 12 a	574 ± 36 a	513 ± 75 a	718 ± 98 a	788 ± 97 a	690 ± 203 a	628 ± 187 a	523 ± 59 a	3525.03 ± 501 c	3224 ± 169 bc	2713 ± 142 b	***

Different letters within a row indicate significant differences according to Tukey’s test (*** *p* < 0.001).

**Table 5:** Values of significance determined with factorial ANOVA performed on production factors and their interactions on content of volatile compounds detected in CS samples.

	Species (S)	Treatment (T)	Roasting (R)	S × T	S × R	T × R	S × T × R
Pyrroles	0.000	0.000	0.625	0.000	0.437	0.293	0.000
Pyrazines	0.287	0.000	0.957	0.000	0.319	0.639	0.219
Furans	0.000	0.000	0.210	0.000	0.000	0.000	0.000
Pyridines	0.000	0.000	0.121	0.000	0.037	0.003	0.000
Sulphur compounds	0.000	0.000	0.000	0.000	0.020	0.001	0.000
Aromatics	0.010	0.000	0.209	0.000	0.655	0.101	0.001
Ketones	0.000	0.000	0.112	0.607	0.909	0.265	0.022
Terpenoids	0.000	0.000	0.000	0.000	0.000	0.000	0.000
Aldehydes	0.000	0.009	0.025	0.537	0.296	0.035	0.000
Alcohols	0.000	0.000	0.013	0.000	0.660	0.010	0.003

**Table 6:** Chromatographic and spectroscopic characteristics (retention times, Rt; UV maxima, λmax; negative pseudomolecular ions, [M-H]^−^; product ions, MS/MS) of phenolic acids detected in CS samples.

Peak	Rt (min)	λmax (nm)	[M-H]^−^ (*m/z*)	MS/MS (*m*/*z*)	Identification
1	6.01	324	353	191, 135, 179	3-*O*-CQA °
2	6.46	325	353	191, 179, 135	5-*O*-CQA *
3	6.56	325	353	173, 179, 191, 135	4-*O*-CQA °
4	8.59	324	367	191, 173	FQA1 °
5	9.04	325	367	191	FQA2 °
6	13.52	327	349	-	di-CQA1 °
7	16.42	326	515	353, 335, 173, 179, 191, 135	di-CQA2 °

CQA: caffeoylquinic acids; FQA isomers: feruloylquinic acids; di-CQA isomers: dicaffeoylquinic acid; -: not present; * comparison with reference standards; ° tentative identification with literature data.

**Table 7:** Content (mg/g; mean ± standard deviation) of phenolic compounds detected in CS samples according to species, post-harvest treatment, and roasting intensity, and results of ANOVA with Tukey’s test for mean comparison.

Post-Harvest Treatment	Washed						Natural						
Species	*Canephora*			*Arabica*			*Canephora*			*Arabica*			
Roasting Degree	Light	Medium	Dark	Light	Medium	Dark	Light	Medium	Dark	Light	Medium	Dark	Significance
3-*O*-CQA	ND	ND	ND	ND	0.17 ± 0.01 a	0.28 ± 0.03 b	ND	ND	ND	ND	ND	ND	***
5-*O*-CQA	0.53 ± 0.22 a	0.48 ± 0.25 a	0.41 ± 0.07 a	1.64 ± 0.35 c	2.16 ± 0.43 d	2.96 ± 0.63 e	0.42 ± 0.23 a	0.41 ± 0.22 a	0.48 ± 0.26 a	1.11 ± 0.33 b	0.61 ± 0.04 a	0.95 ± 0.31 b	***
4-*O*-CQA	0.11 ± 0.01 ab	0.08 ± 0.00 a	0.08 ± 0.01 a	0.21 ± 0.03 c	0.35 ± 0.01 d	0.50 ± 0.04 e	0.10 ± 0.00 ab	0.10 ± 0.00 ab	0.12 ± 0.00 ab	0.14 ± 0.00 b	0.12 ± 0.01 ab	0.11 ± 0.02 ab	***
FQA1	0.05 ± 0.01 ab	0.10 ± 0.00 e	0.05 ± 0.01 abc	0.06 ± 0.00 bcd	0.06 ± 0.00 cd	0.06 ± 0.00 bcd	0.05 ± 0.00 bc	0.07 ± 0.00 d	0.04 ± 0.01 a	0.06 ± 0.00 bc	0.05 ± 0.00 abc	0.06 ± 0.00 bcd	***
FQA2	0.20 ± 0.03 c	0.20 ± 0.01 cd	0.19 ± 0.03 c	0.17 ± 0.01 bc	0.24 ± 0.01 de	0.31 ± 0.01 f	0.27 ± 0.01 ef	0.25 ± 0.00 e	0.30 ± 0.01 f	0.13 ± 0.01 ab	0.10 ± 0.01 a	0.14 ± 0.00 ab	***
di-CQA1	0.18 ± 0.02 ab	0.33 ± 0.02 e	0.19 ± 0.03 abcd	0.37 ± 0.00 e	0.54 ± 0.03 f	0.72 ± 0.04 g	0.25 ± 0.01 d	0.22 ± 0.01 bcd	0.24 ± 0.01 cd	0.18 ± 0.00 abc	0.14 ± 0.02 a	0.20 ± 0.01 abcd	***
di-CQA2	0.15 ± 0.02 b	0.41 ± 0.01 g	0.16 ± 0.03 b	0.23 ± 0.01 c	0.38 ± 0.01 fg	0.45 ± 0.03 h	0.32 ± 0.01 de	0.29 ± 0.00 d	0.35 ± 0.01 ef	0.16 ± 0.00 b	0.08 ± 0.01 a	0.16 ± 0.00 b	***

Different letters within a row indicate significant differences according to Tukey’s test (*** *p* < 0.001); ND—not detected. CQA: caffeoylquinic acid; FQA: feruloylquinic acid; di-CQA: dicaffeoylquinic acid.

**Table 8:** Values of significance determined using factorial ANOVA performed on values of production factors and their interactions for the content of phenolic compounds detected in CS samples.

	Species (S)	Treatment (T)	Roasting (R)	S × T	S × R	T × R	S × T × R
5CQA	0.000	0.000	0.000	0.000	0.000	0.000	0.000
4CQA	0.000	0.000	0.000	0.000	0.000	0.000	0.000
FQA1	0.104	0.000	0.000	0.185	0.000	0.000	0.013
FQA2	0.000	0.001	0.000	0.000	0.000	0.000	0.000
Di-CQA1	0.000	0.000	0.000	0.000	0.000	0.000	0.000
Di-CQA2	0.000	0.000	0.000	0.000	0.000	0.000	0.000

**Table 9:** Values (mean ± standard deviation) of total phenolic content (TPC; mg GAE*/*g of dry powder), radical scavenging activity (RSA; µM TE*/*g of sample), and oxygen radical absorbance capacity (ORAC; µM TE*/*g of sample) detected in CS samples according to species, post-harvest treatment, and roasting intensity. The results of variance analysis with Tukey’s test for mean comparison are shown.

			TPC	RSA	ORAC
Washed	*Canephora*	Light	7.88 ± 0.21 ab	36.47 ± 2.83 a	291 ± 15 bcdef
		Medium	8.20 ± 0.33 bc	37.49 ± 1.10 abc	283 ± 13 bcde
		Dark	8.09 ± 0.07 ab	36.61 ± 2.1 ab	255 ± 15 abc
	*Arabica*	Light	7.14 ± 0.08 a	38.22 ± 1.49 abc	226 ± 15 a
		Medium	8.35 ± 0.53 bc	43.83 ± 0.38 de	248 ± 13 ab
		Dark	9.18 ± 0.32 c	48.33 ± 2.16 ef	263 ± 20 abcd
Natural	*Canephora*	Light	10.30 ± 0.45 d	41.27 ± 1.99 bcd	298 ± 20 cdef
		Medium	10.79 ± 0.49 de	41.61 ± 1.29 cd	306 ± 24 defg
		Dark	11.48 ± 0.29 ef	39.52 ± 0.80 abcd	328 ± 16 efgh
	*Arabica*	Light	12.92 ± 0.36 gh	49.24 ± 1.75 f	360 ± 16 h
		Medium	11.94 ± 0.19 fg	47.74 ± 0.57 ef	337 ± 17 fgh
		Dark	13.39 ± 0.44 h	50.39 ± 0.47 f	348 ± 14 gh
Significance			***	***	***

Different letters within a column indicate significant differences according to Tukey’s test (*** *p* < 0.001).

**Table 10:** Values of significance determined with factorial ANOVA performed on production factors and their interactions on TPC and antioxidant activity in CS samples.

	Species (S)	Treatment (T)	Roasting (R)	S × T	S × R	T × R	S × T × R
ORAC	0.538	0.000	0.717	0.000	0.428	0.257	0.001
TPC	0.000	0.000	0.000	0.000	0.020	0.005	0.000
RSA—DPPH	0.000	0.000	0.004	0.121	0.000	0.001	0.022

## Data Availability

Not applicable.
